# Applying artificial intelligence to predict falls for inpatient

**DOI:** 10.3389/fmed.2023.1285192

**Published:** 2023-11-20

**Authors:** Ya-Huei Chen, Jia-Lang Xu

**Affiliations:** ^1^Department of Nursing, Taichung Veterans General Hospital, Taichung, Taiwan; ^2^Big Data Center, National Chung Hsing University, Taichung, Taiwan

**Keywords:** inpatient falls, artificial intelligence, machine learning methods, XGBoost, EHR data

## Abstract

**Objective:**

Falls are adverse events which commonly occur in hospitalized patients. Inpatient falls may cause bruises or contusions and even a fractures or head injuries, which can lead to significant physical and economic burdens for patients and their families. Therefore, it is important to predict the risks involved surrounding hospitalized patients falling in order to better provide medical personnel with effective fall prevention measures.

**Setting:**

This study retrospectively used EHR data taken from the Taichung Veterans General Hospital clinical database between January 2015 and December 2019.

**Participants:**

A total of 53,122 patient records were collected in this study, of which 1,157 involved fall patients and 51,965 were non-fall patients.

**Primary and secondary outcome measure:**

This study integrated the characteristics and clinical data of patients with falls and without falls using RapidMiner Studio as an analysis tool for various models of artificial intelligence. Utilization of 8 differ models to identify the most important factors surrounding inpatient fall risk. This study used the sensitivity, specificity, and area under the ROC curve to compute the data by 5-fold cross-validation and then compared them by pairwise t-tests.

**Results:**

The predictive classifier was developed based upon the gradient boosted trees (XGBoost) model which outperformed the other seven baseline models and achieved a cross-validated ACC of 95.11%, AUC of 0.990, F1 score of 95.1%. These results show that the XGBoost model was used when dealing with multisource patient data, which in this case delivered a highly predictive performance on the risk of inpatient falls.

**Conclusion:**

Machine learning methods identify the most important factors regarding the detection of inpatients who are at risk of falling, which in turn would improve the quality of patient care and reduce the workloads of the nursing staff when making fall assessments.

## 1 Introduction

There are two important clinical indicators of care: the falls indicator and the pressure injury indicator (1, 2), most of these studies have looked at the causes of the events prior to their occurrence (3). However, Patient falls which occur during hospitalization can cause serious injury and are one of the most difficult patient safety issues that hospitals face. When a patient falls, family members often feel that the hospital staff has not properly fulfilled its care responsibilities, while the hospital feels both aggrieved and powerless. Therefore, preventing patients from falling during hospitalization has always been the most concerning issue within every hospital (4, 5). According to statistical analysis of the Taiwan Patient Safety Reporting System (TPR), 1 out of every 4 medical safety incidents involves a fall. In 2018, the number of falls reported by various medical institutions reached as high as 17,360 patients, the incidence of falls is 0.6%. That statistic translates into 2 patients falling every hour, with the ranking of this safety concern in hospitals being only lower than drug-related incidents (6). Inpatients who fell and caused injuries to themselves extended their length of hospital stay by 6.4 days when compared with those who did not fall. In turn, medical care costs increased for them by 18,257 Taiwan New Dollars (TWDs), causing an annual increase in medical care costs to reach as much as 300 million TWDs (6, 7).

In the United States, approximately 1 million patients fall in hospitals each year, with approximately one-third of those falls considered preventable (8). Therefore, if high-risk groups and fall risk factors can be screened early, this preventative approach could provide both the necessary measures and educational tools needed for medical personnel. This early screening process would help to reduce both the incidence of falls and injury rates of inpatients while also eliminating any subsequent medical costs (9). Presently, many fall risk assessment scales are being used in medical care institutions worldwide, such as the St. Thomas’s risk assessment tool in falling elderly inpatients (STRATIFY) and Hendrich’s High-Risk Fall Model (10–12), which require manual assessment. However, these tools require a clinician’s time for performing the assessment and have a low specificity, which makes it difficult to determine how to focus on fall prevention tactics in a hospital setting (13).

Compared with Western societies, the nurse–patient ratios had a high percentage in Taiwan. Hence, applying machine learning models will increasingly assist in early disease diagnoses and targeted prevention in the medical fields (14). The use of these models is a powerful technique that can accurately predict clinical outcomes and identify important predictors. Artificial intelligence (AI) offers tremendous potential as a tool for improving both safety and predictive performance. Future advances in computing technology will be able to increase the application of electronic health records (EHRs) and electronic administrative data in order to better identify hospitalized patients who are at risk of falling.

Clinicians usually spend a lot of time using fall assessment tools to assess high-risk patients for falls. Moreover, the few features which are captured in these assessments focus primarily on intrinsic risk factors. Several studies have reported predicting high-risk falls using various machine learning algorithms, including the decision tree (10), logistic regression, linear discriminate analysis, naive bayes, kernel support vector machine, random forest, and neural networks (12, 15, 16). However, few studies have shown an accuracy rate of at least 80%. In addition, most studies have been based on Western populations with a limited number of samples.

Since most fall risk assessments in the past have been performed manually and caregivers have only been able to communicate patients’ fall risk, this study applies an automated machine learning approach to help better identify important inpatient fall risk factors in Taiwan, and to validate the predictive efficacy of the model on a training and testing dataset. The goal of this study is to utilize artificial intelligence to predict who is at high risk of falling in hospitalized patients, while also replacing the use of manual fall assessment tools. Additionally, this study was able to demonstrate the effectiveness of the XGBoost model when working with multi-source patient data, in which case the model could provide higher prediction performance for inpatient fall risk. However, using machine learning methods does have the potential to identify the most important factors for detecting inpatients at risk of falling, thereby improving the quality of patient care and reducing the workload of caregivers when performing fall assessments. This study may provide a reference for the development of AI-based fall prediction models for hospitalized patients.

## 2 Materials and methods

### 2.1 Ethical approval

This study was approved by the Institutional Review Board of Taichung Veterans General Hospital (IRB No. CE20256B). All the data were anonymized data, and informed consent was hence waived.

### 2.2 Patient and public involvement

This research was performed without patient involvement. Patients were not invited with regards to design of study, measurement of outcome, and interpretation of results.

### 2.3 Study setting, design, and ethical considerations

This study retrospectively used EHR data taken from the Taichung Veterans General Hospital clinical database between January 1, 2015 and December 31, 2019, for patients who were at least 20 years of age. An exclusion conditions condition was that the collected data would not include the Hospice Center of the hospital, because the medical records of the Hospice Center are incomplete. Due to the government’s emphasis on falls and the promotion of the Taiwan Patient Safety Reporting System, the chance of unreported or unidentified falls during data collection is very rare and less than 0.05. A total of 53,122 patient records were collected for this study, of which 1,157 involved fall patients and 51,965 did not. A review of the available literature shows that many manuscripts use the Morse Fall Scale to measure the risk of falls, which has been demonstrated to be a reliable method. However, this study sought to know whether patient characteristics and clinical data could each be useful in predicting falls and non-falls. This case–control study collected fall and non-fall patient characteristics and clinical data during hospitalization. In this study, since this nursing data processing is very important, this study was conducted manually to find out whether the data of 46 eigenvalues were correct or not. The data cleaning process was used to manually process the data in order to exclude unreasonable data such as, BMI > 80, blood pressure < 30, respiration >100, heart rate < 10, body temperature > 50, along with others.

### 2.4 Model training

This study uses the RapidMiner Studio version 9.8 Enterprise edition as an analysis tool for various models of artificial intelligence. RapidMiner Studio is a visual analysis process design software, which allows analysts to fully understand the process, where the results taken from the software can be used with full confidence. The purpose of using this tool is to quickly assist in the training mode phase. This study tested the performance of various models in order to find the ideal model for use in the prediction phase. Overall, this study tried to use the eight models, the Naive Bayes (NB), Generalized Linear Model (GLM), Logistic Regression, Decision Tree (DT), Random Forest (RF) (17, 18), XGBoost and Neural Network (NN). This study adopted the method of upsampling for the recession group, attempting to obtain as much as possible of two types of data in order to achieve a balance for training the model. This study used the Bayesian optimization strategy to obtain the hyperparameters on the eight models used in the training, and used the 5-fold cross-validation process to train the model. Finally, in order to ensure the usability of the model, we chose the accuracy as the most valuable indicator of the classification model, the sensitivity and specificity as the most commonly used indicators in the medical field, the AUC as the predictive ability of the model (19), the higher the index means that the model predicts well, and the F1-score as the indicator calculated by considering both the precision rate and the recall rate.

### 2.5 Statistical analyses

Basic demographic data, biochemical examinations, and continuous variables are shown as the mean (standard deviation, SD), with categorical variables shown as the number (percent). The Mann–Whitney U test and Chi-square test were used to compare variables between the nonfaller and faller groups. All data were tabulated and analyzed using Microsoft Excel 2010 and SPSS for Windows, version 21. Statistical significance was set at a *p* value lower than 0.05 for all tests.

## 3 Results

This study identified a total of 1,157 hospitalized patients who had fallen and 51,965 patients who had not during their hospitalization periods. The definition of “fall” is: an unexpected change in body position, when where the center of gravity is out of balance, one cannot make a timely and effective response, causing the whole body to collapse and fall to the ground or a lower place (20).

After screening for candidate variables using the chi-square test or Mann–Whitney U test, 46 features were selected for the machine learning model, as shown in Table 1. Based on Bayesian optimization and 5-fold cross-validations of the training set, the parameters eventually collected for the models included ACC (accuracy), AUC (area under the ROC curve), sensitivity, specificity, and F1 score.

**Table 1:** Baseline characteristics of hospitalized patients.

Features	Non-Fallers (*n* = 51,965)		Fallers (*n* = 1,157)		*p-*value
Age	57.0	±16.0	62.1	±15.4	<0.001**
Gender					<0.001**
Female	26,174	(50.4%)	472	(40.8%)	
Male	25,791	(49.6%)	685	(59.2%)	
Body height	161.7	±8.7	161.1	±8.9	0.064
Body weight	63.9	±13.5	63.1	±13.2	0.053
Body mass index	24.4	±4.5	24.3	±4.6	0.339
Admission method					<0.001**
Walk	35,094	(67.5%)	394	(34.1%)	
Wheelchair	5,664	(10.9%)	245	(21.2%)	
Push the bed	11,205	(21.6%)	518	(44.8%)	
Hold in	2	(0.0%)	0	(0%)	
*Vital signs*					
Systolic blood pressure	133.6	±22.1	133.8	±25.0	0.638
Diastolic blood pressure	78.0	±13.7	77.2	±14.4	0.027*
Mean arterial pressure	96.5	±14.7	96.1	±16.1	0.339
Heart rate	84.4	±15.3	88.8	±17.2	<0.001**
Respiratory rate	18.1	±1.9	18.5	±2.0	<0.001**
Body temperature	36.5	±0.6	36.4	±0.6	<0.001**
Unconscious	1,141	(2.2%)	61	(5.3%)	<0.001**
Insomnia	7,752	(14.9%)	386	(33.4%)	<0.001**
Poor vision	4,648	(8.9%)	150	(13.0%)	<0.001**
Hearing impaired	2,296	(4.4%)	96	(8.3%)	<0.001**
Frequent urination	5,234	(10.1%)	227	(19.6%)	<0.001**
Diarrhea	2,897	(5.6%)	103	(8.9%)	<0.001**
Muscle weakness	131	(0.3%)	8	(0.7%)	0.009**
Assistive devices	3,758	(7.2%)	190	(16.4%)	<0.001**
*Chronic diseases*					
Postural hypotension	0	(0%)	0	(0%)	––
Dementia	177	(0.3%)	2	(0.2%)	0.473
Depression	52	(0.1%)	2	(0.2%)	0.763
Parkinson’s disease	194	(0.4%)	0	(0%)	0.066
Arthritis	1,736	(3.3%)	6	(0.5%)	<0.001**
Cataract	118	(0.2%)	4	(0.3%)	0.601
Glaucoma	94	(0.2%)	1	(0.1%)	0.689
Deafness	0	(0%)	0	(0%)	––
*Special disease*					
Myocardial infarction	473	(0.9%)	2	(0.2%)	0.013*
Arrhythmia	5	(0.0%)	0	(0%)	1.000
Heart failure	1,046	(2.0%)	20	(1.7%)	0.565
Pneumonia	2,117	(4.1%)	35	(3.0%)	0.086
Stroke	10	(0.0%)	0	(0%)	1.000
Epilepsy	188	(0.4%)	3	(0.3%)	0.743
Anemia	917	(1.8%)	36	(3.1%)	0.001**
*Surgery*					
Spine surgery	895	(1.7%)	16	(1.4%)	0.444
Lower limb surgery	2,094	(4.0%)	62	(5.4%)	0.028*
*Drug type*					
Hypnotic	9,238	(17.8%)	640	(55.3%)	<0.001**
Psychiatric	6,440	(12.4%)	551	(47.6%)	<0.001**
Diuretics	9,802	(18.9%)	523	(45.2%)	<0.001**
Antiarrhythmic	8,333	(16.0%)	425	(36.7%)	<0.001**
Antihypertensive	14,871	(28.6%)	592	(51.2%)	<0.001**
Morphine analgesics	22,921	(44.1%)	701	(60.6%)	<0.001**
Antiepileptic	5,132	(9.9%)	445	(38.5%)	<0.001**
Foxglove cardiotonic	284	(0.5%)	27	(2.3%)	<0.001**
Hypoglycemic	5,722	(11.0%)	304	(26.3%)	<0.001**
Antihistamine	20,510	(39.5%)	566	(48.9%)	<0.001**
Chemotherapy	8,887	(17.1%)	97	(8.4%)	<0.001**
Pain score					<0.001**
1–3	47,407	(91.23%)	625	(54.0%)	
4–10	4,558	(8.77%)	532	(46.0%)	
Fall history	2,342	(4.5%)	646	(55.8%)	<0.001**

Mann–Whitney *U* test. Chi-Square test. *p < 0.05, **p < 0.01.

As shown in Table 2, when comparing the performance of different imputation methods, the accuracy rate is between 93.74 and 98.91%. The AUC as a measure of the performance of the classifier was between 0.867 and 0.959, while the F1 score was between 49.57 and 69.52%. In terms of AUC, the deep learning methods showed the highest discriminatory ability (0.959), while the GLM performed the best in regards to ACC and specificity. These results may be due to the data being imbalanced, where the proportions of the declining groups were very different from those of the nondeclining groups. Therefore, the upsampling method was adopted for the declining groups, and attempts were made to use the two types of data as much as possible in order to achieve balance.

**Table 2:** Model performance comparison with different machine learning methods.

Machine learning methods	ACC	AUC	Sensitivity	Specificity	F1 Score
Deep learning	98.58%	0.959	66.67%	99.29%	67.10%
Decision tree	98.84%	0.867	55.33%	99.81%	67.49%
Random forest	98.90%	0.957	49.57%	100.00%	66.28%
XGboost	98.44%	0.910	66.28%	99.15%	64.88%
Naive Bayes	93.74%	0.934	70.89%	94.25%	33.02%
Generalized linear model	98.91%	0.953	57.06%	99.84%	69.47%
Logistic regression	98.88%	0.950	58.50%	99.78%	69.52%

RapidMiner was chosen as the tool for data balancing because (1) it was first read in the original data set, (2) it first randomly selected 60% of the data in the undeclined ethnic group, (approximately 31,250 subjects), and (3) it used the Synthetic Minority Oversampling Technique (SMOTE) (4, 5). The SMOTE method was used to upsample the descended ethnic groups, where it generated the 31,250 pieces of data from the descending ethnic groups for output used in model training. As shown in Table 3, the area under the receiver operating characteristic (ROC) curves, sensitivity, specificity, accuracy, and F1 scores were all compared and analyzed between the training sets. XGBoost achieved the best performance among the machine learning models. The ACC, sensitivity, specificity and F1 scores in the training set were 95.11, 95.37, 94.86 and 95.10%, respectively. This study selected the 25 best prediction features for retraining the XGBoost model where the training set sensitivity was 95.37%, with a specificity of 95.37% and an ACC of 0.99.

**Table 3:** Model performance comparison with different machine learning methods after data balancing.

Machine learning methods	ACC	AUC	Sensitivity	Specificity	F1 Score
Deep learning	91.26%	0.971	90.67%	91.84%	91.2%
Decision tree	92.41%	0.965	92.28%	92.54%	92.4%
Random forest	91.08%	0.968	87.83%	94.3%	90.8%
XGboost	95.11%	0.990	95.37%	94.86%	95.1%
Naive Bayes	86.91%	0.949	88.97%	84.85%	87.18%
Generalized linear model	90.93%	0.966	88.34%	93.51%	90.69%
Logistic regression	91.23%	0.968	88.91%	93.56%	91.02%

## 4 Discussion

From the years 2016 to 2019, according to Taiwan Medical Center, the incidence rate of falls in Taiwan hospitals was about 0.6% (21). In our hospital, the incidence rate of falls was only 0.4%. Reducing the incidence and injury rates of falls has always been an important responsibility with regards to patient safety in our hospital. Therefore, this study has aimed to use machine learning methods to better identify the most important factors necessary in the detection of inpatients who are at risk of falling. This study can both improve the quality of patient care, and reduce the workload of nursing staff by improving fall assessment methods. This method gives clinicians more time to prepare and prevent patients from falling. It will also decrease the number of injuries in patients due to falls, reduce the length of hospital stays by 6.4 days, and save up to 18,257 million TWD per inpatient in health care costs (6, 7).

Fall risk assessment tools were developed as part of an evidence-based fall safety initiative. For instance, Chen et al. (6) used Taiwan’s National Health Interview Survey to develop an elderly fall risk assessment plan and then verified fall predictive factors through the survey. A total score higher than 6 shows a high risk, with a sensitivity of 75.16% and a specificity of 52.75% in detecting falls. In addition, after studying the factors related to patient falls in Taiwan in the year 2001, some researchers found that there were six risk factors; agitation, poor vision, poor walking frequent urination/diarrhea, fall history, dizziness, and drug-related causes.

In addition to dizziness, drug-related falls combined with STRATIFY and the Hendrich II all risk model, which involves 14 self-developed risk factor assessment scales, the risk factors include being a male an age greater than 65 years (9, 21). Recently, in the “Balance Assessment and Fall Prevention Care and Guidance Guidelines,” formulated by the Ministry of Health and Welfare in 2019, it has been recommended that health care professionals use fall risk assessment to systematically and comprehensively identify factors that increase the risk of falls for the elderly, while also developing an individualized fall prevention program (9).

This study applied 46 eigenvalues that were reported related to the contribution to the prediction of falls. Among these features, pain score, fall history, hypnotics, diuretics, and osteoarthritis were the most relevant factors correlated with inpatient falls (10, 12, 16). While some of these factors have been identified as predictors of patient falls in previous studies, there is still room to learn whether a patient’s cognition test, Mini-mental state exam score and Morse Fall scale assessment are all valid predictors of falls when hospitalized in a medical center or surgical nursing unit. It is worth noting that existing fall risk assessments do not contain all the items identified in our report regarding important features surrounding falls. As a result, an accurate prediction model that integrates simple and interpretable assessment tools involving high-performance contemporary machine learning methods could provide valuable clinical decision support in the medical field, enhance the quality of patient care, and reduce the workload of nursing staff when making fall assessments.

This study used the SMOTE method to insert a small number of samples to artificially synthesize new samples and add them in to the data set. This causes the problems of overlap between classes (overlapping) and oversampling (oversampling), where technology modifies unequal data classes to create a balanced data set, obtaining the advantage of having no information loss while reducing both oversampling and overfitting (20).

This study applied to machine learning methods that were able to determine the major predictive factors surrounding hospitalized patient falls, while also identifying the most important risk factors related to inpatient falls. Additionally, the study applied cross-validated prediction models from EHRs, as well as administrative data that identified the risk of falls based on that easily obtainable clinical data. This study also used a prediction model for patients who fell during hospitalization with a relatively large sample size according to the machine learning algorithms and electronic health records. This study found that the XGBoost algorithm achieved the best performance among seven machine learning models. The sensitivity value of the XGBoost model was 95.37%, with a specificity of 94.86% in the training set. The AUC and ACC were 0.99 and 95.11%, respectively. This prediction model has the potential to assist healthcare providers and organizational leadership decision-making which would improve the quality of care provided to patients.

This study be able to determine that, the XGBoost model showed an outstanding ability to solve overfitting, imbalanced samples and misclassification cost issues through regularization and pruning strategies (22). The study involved an optimized combination of decision tree algorithms and linear regression analyses under a gradient boosting framework (22). The XGBoost model was able to effectively reduce the irrelevant features. Moreover, the XGBoost classifier model was easier to extend to include new views of data, as this study just needed to train subclassifiers of the model on the new data rather than retraining the entire model from scratch.

Finally, this study implemented the trained model immediately in the clinic with limited patient information including age, gender, ethnicity and diagnosis. The predicted outcomes are presented on a dashboard and ward nursing staff can be informed more quickly of those patients who require special care, so the available advantage in serious falls prevention depends on the lead time of the assessment, which is important in situations such as deliberate falls and behavioral falls. In practice, the XGBoost model can be used in the clinical setting to predict severity after fall assessment using one of the fall risk assessment tools mentioned above (e.g., the Morse Falls Scale (MFS) (23), the STRATIFY Scale), and the Hester-Davies Scale (24). This additional layer of alerts for healthcare providers will allow for more efficient and cost-effective implementation, while also reducing the time required to prevent falls.

## 5 Limitations

This study has certain limitations. Although it offers advantages over existing fall risk assessment tools, the XGBoost model would need certain advantages. First, one a subset of inpatient data for the period from January 2015 to December 2019 was extracted from the nursing record system and adverse event reporting system of the Taichung Veterans General Hospital. Testing our models on data taken from other hospitals would still be needed to establish external validity. Second, this study was conducted within Taichung Veterans General Hospital, and our model requires validation of its accuracy through clinical testing. Although this study identified models that have a relatively stable performance, sensitivity, specificity ACC and ROC, the estimates were subject to a case–control study design. The model performance tests would be best performed with a population sample; hence our ongoing study is continuing to conducting a validation of the XGBoost model in hospitalized patients for a period of one year. The XGBoost tool will be used to predict a high risk of fall injuries for fall patients, which will show the true calibration and discrimination of XGBoost.

## 6 Conclusion

Falls are an important issue in Taiwan’s medical institutions, and hospitals have a well-established patient safety reporting system in place. This study showed that the XGBoost model is a useful tool for prediction because it achieved an accuracy of 95.11%, and the use of machine learning methods has the potential to identify the most important factors to be considered when detecting the risk of falls in hospitalized patients. The clinical application of this study can be faster for the caregivers to know that there should be fall patients as those, therefore this model and application can be effective in improving the quality of personalized care and also reduce the workload of the caregivers in performing fall assessment. Therefore, this study can be used as a future reference to better promote the development of fall prevention interventions.

## Data availability statement

The raw data supporting the conclusions of this article will be made available by the authors, without undue reservation.

## Ethics statement

The studies involving humans were approved by the Institutional Review Board of Taichung Veterans General Hospital. The studies were conducted in accordance with the local legislation and institutional requirements. Written informed consent for participation was not required from the participants or the participants’ legal guardians/next of kin because This research was performed without patient involvement. Patients were not invited with regards to design of study, measurement of outcome, and interpretation of results.

## Author contributions

Y-HC and J-LX: Data acquisition. J-LX: Supervision or Mentorship. Y-HC had full access to all the data in the study and takes responsibility for the integrity of the data and the accuracy of the data analysis.
